# The Canadian Neighbourhood Early Childhood Development (CanNECD) Socioeconomic Index: Stability and Measurement Invariance Over Time.

**DOI:** 10.23889/ijpds.v7i3.2048

**Published:** 2022-08-25

**Authors:** Barry Forer, Molly Pottruff, Eric Duku, Martin Guhn, Magdalena Janus

**Affiliations:** 1 The University of British Columbia; 2 McMaster University

## Objectives and Approach

The CanNECD SES Index is a composite of 10 Canadian Census and Income Tax Filer variables, aggregated to 2,038 custom neighbourhoods covering all of Canada. The baseline 2006 Index accounted for 32% of the neighbourhood-level variance in overall developmental vulnerability in Kindergarten children, as measured by the Early Development Instrument (EDI). Other existing SES indices accounted for 17% at most.

The Index now has two additional time points (2011 and 2016), which allows an evaluation of its consistency over time. Our objective is to assess three aspects of the Index’s temporal consistency. The first is the consistency of the strength of association between the Index and vulnerability rates across EDI developmental domains. The second is the consistency of neighbourhoods’ quintile rank over time. Finally, we use Confirmatory Factor Analysis in an SEM framework to assess the Index’s measurement invariance over time.

## Results

For each EDI domain, the strength of association between Index scores and neighbourhood-level vulnerability rates were either maintained or minimally declined over time. Additionally, neighbourhood quintile rankings were highly consistent over time with over 60% of neighbourhoods in the same quintile between 2006 and 2016, and fewer than 3% with a greater than one-quintile change. Finally, our preliminary measurement invariance results show at least configural invariance over the three time points.

## Conclusion / implications

Our results confirm the stability of the CanNECD Index, justifying its utility for: mapping SES indicators across neighbourhoods and over time, contextualizing neighbourhood-level developmental vulnerability in young children, and identifying interesting neighbourhoods for future study, especially those where the children are faring much better than predicted by the Index.

